# BUSnet: A Deep Learning Model of Breast Tumor Lesion Detection for Ultrasound Images

**DOI:** 10.3389/fonc.2022.848271

**Published:** 2022-03-25

**Authors:** Yujie Li, Hong Gu, Hongyu Wang, Pan Qin, Jia Wang

**Affiliations:** ^1^Faculty of Electronic Information and Electrical Engineering, Dalian University of Technology, Dalian, China; ^2^Department of Surgery, The Second Hospital of Dalian Medical University, Dalian, China

**Keywords:** breast ultrasound, lesion detection, deep learning, unsupervised pre-processing, bounding-box regression

## Abstract

Ultrasound (US) imaging is a main modality for breast disease screening. Automatically detecting the lesions in US images is essential for developing the artificial-intelligence-based diagnostic support technologies. However, the intrinsic characteristics of ultrasound imaging, like speckle noise and acoustic shadow, always degenerate the detection accuracy. In this study, we developed a deep learning model called BUSnet to detect the breast tumor lesions in US images with high accuracy. We first developed a two-stage method including the unsupervised region proposal and bounding-box regression algorithms. Then, we proposed a post-processing method to enhance the detecting accuracy further. The proposed method was used to a benchmark dataset, which includes 487 benign samples and 210 malignant samples. The results proved the effectiveness and accuracy of the proposed method.

## Introduction

Breast cancer is the most frequently diagnosed among Chinese women (1). Early detection of breast cancer is an effective method to decrease the morality rate dramatically (2). Because the ultrasound imaging (US) technique is a low-cost way to offer favorable sensitivity and detection rates for early cancer, it is a widely applied modality for breast cancer detection in China (3). Unlike computed tomography or magnetic resonance imaging, quality control is a critical issue for US. Especially when handheld ultrasound is used to screen the whole breast, the imaging quality is positively related to the skill levels of the radiologists (4). In addition, the artifacts in US, including the speckle noise and the acoustic shadow, are another issue degenerating the imaging quality. Thus, it is imperative to develop US diagnostic support technologies to resolve these operation-dependent difficulties.

Over the past decade, machine learning (ML) is being increasingly applied in the research and application of medical imaging techniques (4, 5). Furthermore, deep learning (DL) is an advantage subset of ML, in which convolutional neural networks (CNN) were initially designed for image analysis (6). The state-of-the-art algorithms of ML and DL are extensively applied for image classification (6), object detection (7), and segmentation (8), which offers the potential to develop US diagnostic support technologies.

Classification estimates the label for the entire image (9). constructed CNN to classify the US images into benign and malignant breast tumors. CNN was further improved by introducing a matching layer for breast mass classification (10). The support vector machine, VGG, and ResNet were also applied to the classification issues for the breast US images (11–13). Object detection can automatically identify the specified targets in the images (12). used VGG and ResNet for the classification and lesion detection for US images (14). evaluated the performance of detecting breast cancer lesions in US images for three deep learning models. Segmentation is used for the further precise measurements and structures of targets (15). proposed a U-net-based segmentation algorithm for suspicious breast masses in US images. Object detection can offer more detailed information than classification (16) and can also save more costs of labeling data and training networks than segmentation (17). For these reasons, object detection is often used for the breast disease lesion identification for US images, which can help the radiologist make a diagnosis efficiently with expected high sensitivity and specificity (14).

Although several ingenious methods for the breast tumor lesions detection for US images have been reported, as indicated by (4), the progress of medical artificial intelligence (AI) research and development in US is slower than that in other modalities. This is mainly because a single ML or DL method cannot tackle the problem caused by the artifacts in US images. Effective ML and DL methods for lesion detection for US images should integrate preprocessing, like denoising, and post-processing, like redundancy reduction. However, such integration has not been considered enough in the previous work. In this research, we proposed a breast tumor lesion detection method called BUSnet, which includes a preprocessing procedure, a deep learning model for the lesion detection, and a post-processing procedure. In the preprocessing procedure, we combined the unsupervised Canny edge detection (18) with the selective search (19) to determine the region of interest (RoI) candidates. We trained a G-CNN (20) for the bounding-box regression. We proposed a novel post-processing method by improving the non-maximum suppression method (21) to enhance the detection accuracy. The dataset obtained from (22) was used to demonstrate the outstanding performance of our method.

## Methods

### Data

**Figure 1:**
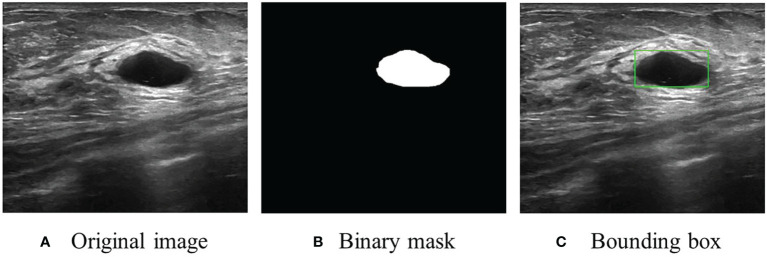
An US image sample. **(A)** Original image. **(B)** Original segmentation ground truth in binary mask form provided by (22). **(C)** Conversion to bounding box as the ground truth for RoI detection and localization.

The dataset, including 780 samples (133 normal samples, 487 benign samples, and 210 malignant samples), was achieved from (22). In this research, the benign and malignant samples were used to develop BUSnet. 80% of samples were used as the training data, and the rest were used as the test data. A sample example is shown in **Figure 1**.

### Algorithm Framework

**Figure 2:**
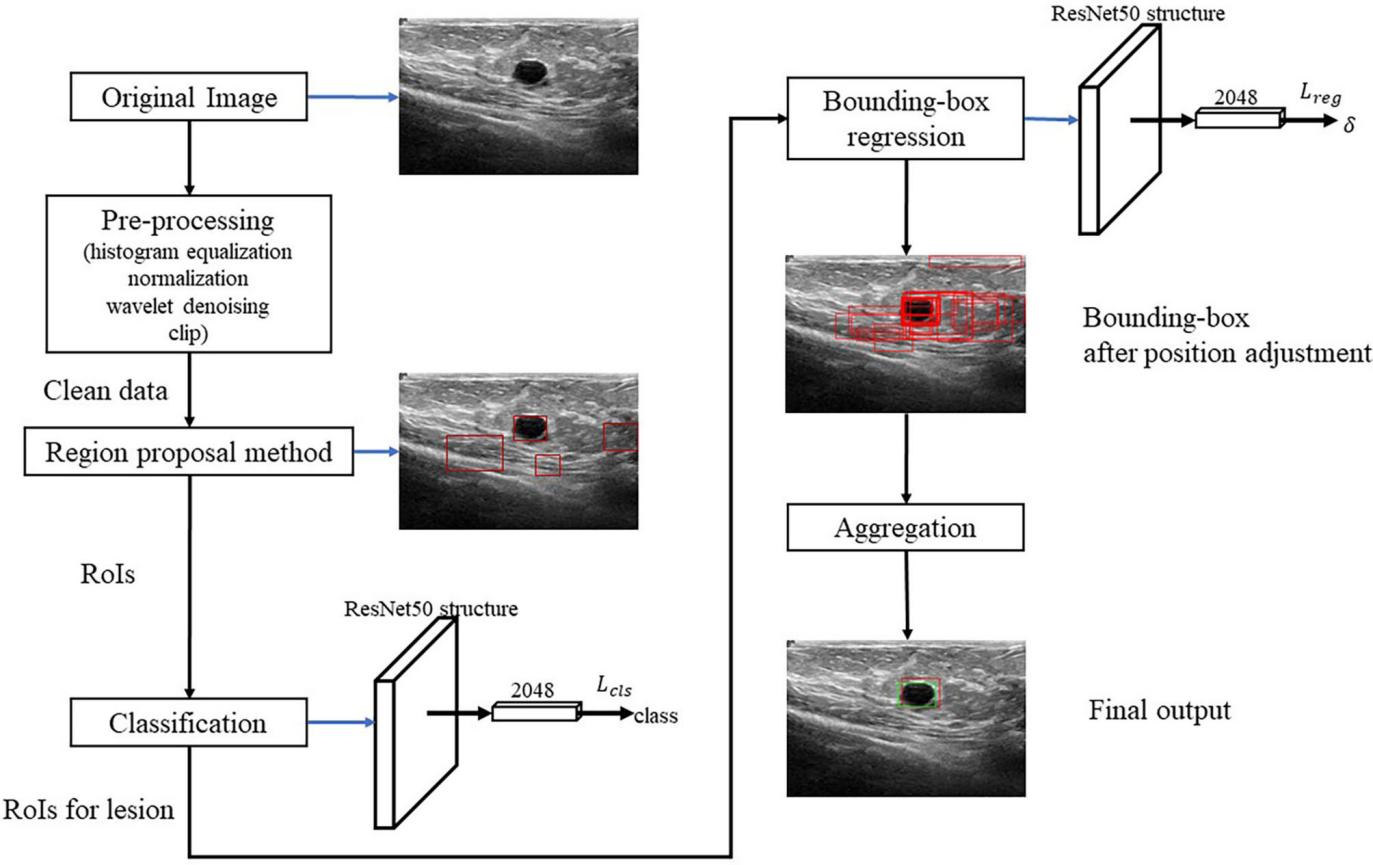
Framework of BUSnet.

**Figure 2** shows the algorithm. RoI candidates are screened out by a preprocessing method and an unsupervised region proposal method. The classification and bounding-box regression networks were constructed for the RoI candidates. We select all the RoI candidates with the probability of being lesion higher than 0.9 according to (23) and adjust their position with bounding-box regression. Finally, we aggregate these bounding boxes and achieve the final output.

### Pre-Processing

We first conduct the histogram equalization, the normalization, and the wavelet-based denoising to the US images to ease the effect of the uncertainties like the speckle noise. Then, we clip 30% lower part of the images to reduce the redundant RoIs according to (24).

### Region Proposal and Classification

We propose an unsupervised region proposal method by using the Canny edge detector and the selective search to obtain the RoI candidates. The Canny edge detector is carried out as follows:

1. The Gaussian-smoothing is applied to the images.
2. The Sobel operators in two directions

${\text{Sobel}}_{x}=\left(\begin{array}{lll} 1 & 2 & 1\\ 0 & 0 & 0\\ -1 & -2 & -1 \end{array}\right),{\text{ Sobel}}_{y}=\left(\begin{array}{lll} 1 & 0 & -1\\ 2 & 0 & -2\\ 1 & 0 & -1 \end{array}\right)$ .
  are used to the smoothed images to detect the edges.
3. Non-maxima suppression is conducted. The gradient intensity of the current pixel and that of the two pixels along the positive and negative gradient directions are compared. If the gradient intensity of the current pixel is larger than the other two pixels, the current pixel is retained as an edge point; otherwise, the current pixel will be discarded.
4. A two-threshold test is applied. A high threshold and a low threshold are set. If the gradient value of the edge pixel is higher than the high threshold, it will be marked as a strong edge pixel; if the gradient value of the edge pixel is between the two thresholds, it will be marked as a weak edge pixel; if the gradient value of the edge pixel is less than the low threshold, it will be suppressed.

The Canny edge detection removes redundant information and noise. Then, the selective search method is applied to identify RoI candidates. In the selective search method, for the regions *i* and *j*, let *s*_gray_(*i,j*), *s*_texture_(*i,j*), *s*_size_(*i,j*), and *s*_fill_(*i,j*) denote the similarities of gray level, texture, size, and fullness, respectively. The sum *s*(*i,j*) = *s*_gray_(*i,j*) + *s*_texture_(*i,j*) + *s*_size_(*i,j*) + *s*_fill_(*i,j*) is used to measure the total similarity between the regions *i* and *j*. In the following procedure, the total similarity is used to emerge regions:

1. Initialize the region set *R* = {1,2,…,*n*} and similarity set *S* = ∅.
2. Calculate pair-wised total similarities *s*(*i,j*) and conduct *S* = *S* ∪ *s*(*i*, *j*).
3. Let *s*(*p,q*) = max *S*, and *t* = *p* ∪ *q*.
4. Remove all the similarities associated with regions *p* and *q*, i.e., *S* = *S*\{*s*(*p*,*), *s*(*,q)}.
5. Take *t* as a new region and *R* = *R* ∪ *t* and *S* = *S* ∪ {*s*(*t*,*)}.
6. Repeat step 2-5, until *S* = ∅.

After finishing the region proposal, the RoIs are classified into lesion and normal types using ResNet50. For the RoIs with probability larger than 0.9, their position will be further adjusted by using bounding-box regression.

### Bounding-Box Regression

**Figure 3:**
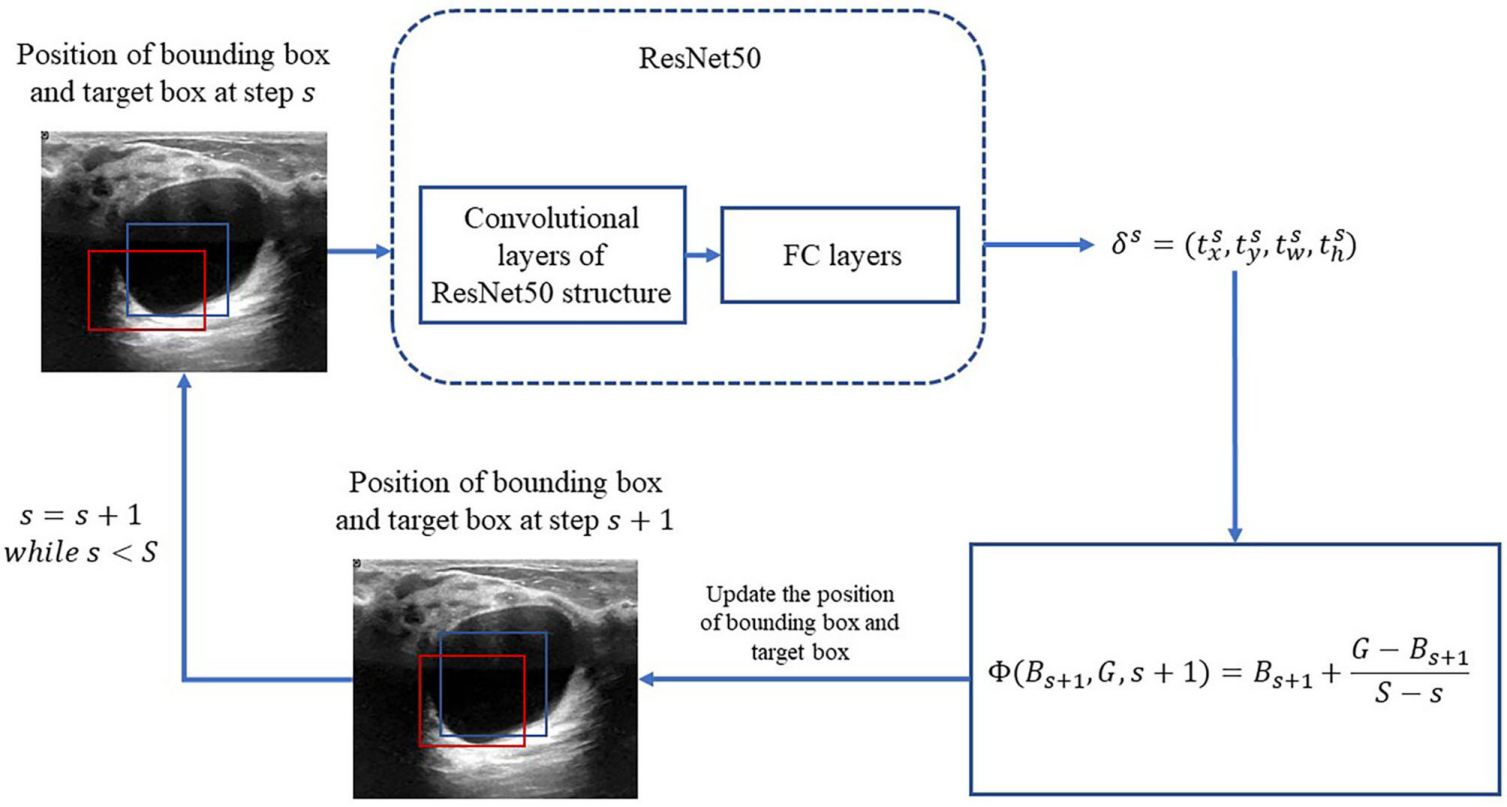
Process of iterative bounding-box regression. The red rectangle represents the bounding box, and the blue one represents the target box. At each step, the network modifies the bounding box, and the target box coordinates and uses the new bounding box as the input.

In the following, the RoIs classified as lesions are denoted as bounding box and used as the input of the bounding-box regression. The procedure is illustrated in **Figure 3**. Because ResNet50 has been proven to be competent for the feature extraction of breast US images (25), we use ResNet50 as the backbone network for the bounding-box regression.

Let *B* = (*x,y,w,h*) denote the bounding box location, in which *x* and *y* are the x-axis and y-axis locations of the upper-left vertex of the box, respectively; *w* and *h* are the width and height of the box, respectively. With respect to these denotations, let *G* = (x*,y*,w*,h*) denote the location of the true region. The error between the bounding box and the truth box *δ* = (*t_x_,t_y_,t_w_,t_h_*) is obtained by the following function:


(1) $$\delta =(t_{x},t_{y},t_{w},t_{h})=\left(\frac{x^{*}-x}{w_{a}},\frac{y^{*}-y}{h_{a}},\log\left(\frac{w^{*}}{w}\right),\log\left(\frac{h^{*}}{h}\right)\right)$$

For convenience, let *δ* = *Δ*(*B,G*) denote the transformation (1) from the locations *B* and *G* to the difference *δ*. Consequently, *G* can be calculated from


(2) $$G=(x^{*},y^{*},w^{*},h^{*})=(t^{*}w+x,t^{*}h+y,w\exp\;(t_{w}^{*}),\;h\;\exp\;(t_{h}^{*}))$$

Let *G* = Γ(*B*, δ) denote the transformation (2) from the bounding box location *B* and the error *δ*.

Motivated by (20), an iterative method is applied for the bounding-box regression. At first, the total iteration *s* is prefixed. For *s* = 1,2,…,,*S*, the target bounding box is obtained by


$$\Phi (B^{s},G,s)=B_{s}+\frac{G-B^{s}}{S-s+1},$$

in which *B^s^* is updated at each iteration as the following:


$$B^{s}=\Gamma (B^{s-1},\delta^{s-1})$$

Then, we construct the loss function for the bounding-box regression as the following:


$$L_{\text{reg}}=\sum_{s=1}^{S}\sum_{i=1}^{N}\left[I\left(B_{i}^{s}\notin B_{\text{bg}}\right)\times L_{1\text{s}}\left(\delta_{i}^{s}-\Delta \left(B_{i}^{s},G_{i},s\right)\right)\right],$$

where *N* is the total of bounding boxes; $B_{i}^{1}$ is the initialization of the *i*th bounding box; and *B*_bg_ is the set of background bounding boxes with the Jaccard index being smaller than 0.2 with respect to the true box, with the definition of the Jaccard index for pixel sets *A* and *B*


$$J(A,B)=\frac{\left|A\cap B\right|}{\left|A\cup B\right|},$$

$\text{I }(B_{i}^{s}\notin B_{\text{bg}})$

is the indicator function

$\text{I }(B_{i}^{s}\notin B_{\text{bg}})=\{\begin{array}{l} 1,\text{if}B_{i}^{s}\notin B_{\text{bg}}\\ 0,\text{ otherwise} \end{array};$

*L*_1_*_s_* is the smooth *l*_1_ loss as the following:

${\text{L}}_{1s}(x)=\{\begin{array}{l} 0.5x^{2},\text{ if |}x|<1\\ |x|-0.5,\text{ otherwise} \end{array}.$

### Aggregation

In the aggregation, the regressive bounding boxes are integrated into one box to eliminate the redundant information. Let ℬ denote the set of all the bounding boxes and initialize index *k* = 0. The aggregation procedure is as follows:

1. If ℬ ≠ ∅, let *k* = *k* + 1. Initialize *A*_k_ = { *B*_k0_} with *B*_k0_ being the box nearest to the center of the image and ℬ = ℬ\{ℬ*_k_*_0_}.
2. For each ℬ ∈ ℬ, calculate pairwise Jaccard indices with the elements in 𝒜*_k_*. If any Jaccard index is larger than 0.5, 𝒜*_k_* = 𝒜*_k_* ∪ {*B*} and ℬ = ℬ\{ℬ}.
3. If ℬ becomes ∅, assume {𝒜*_k_* |*k* = 1,2,…, *K*} is obtained. Let 𝒜^*^ denote the bounding box set with the largest size among {𝒜*_k_*}.
4. Calculate all the pairwise Jaccard indices of the elements in 𝒜^*^. Assume that *B*_1_, *B*_2_ ∈ 𝒜 are the two bounding boxes with the largest Jaccard index. If *J* (*B*_1_, *B*_2_) ≥ 0.7, *B* = *B*_1_ ∩ *B*_2_. If 0.5 ≤ *J*(*B*_1_, *B*_2_) < 0.7, construct the smallest bounding box *B* that can cover *B*_1_ ∪ *B*_2_.
5. 𝒜^*^ = 𝒜^*^\{*B*_1_}, 𝒜^*^ = 𝒜^*^\{*B*_2_}, and 𝒜^*^ = 𝒜^*^∪ *B*.
6. Repeat aggregations 4 and 5, until 𝒜^*^ contains only one bounding box.

Note that multiple 𝒜^*^s can be achieved in step 3. In this case, all the 𝒜^*^s are aggregated by repeating steps 4 and 5. Then, the bounding box with the largest classification confidence is determined as the final aggregated bounding box.

### Performance Metrics

The Jaccard index between the predicted set of lesion pixels and the truth ground, accuracy, precision, recall, and *F*_1_ score are used as the prediction performance metrics. Accuracy, precision, recall, and *F*_1_ score are defined as follows:


$$\text{accuracy}=\frac{TP}{TP+FP+FN},$$

$$\text{precision}=\frac{TP}{TP+FP},$$

$$\text{recall}=\frac{TP}{TP+FN},$$

$$F_{1}\text{ score}=\frac{2recall\times precision}{recall+precision},$$

where *TP* is true positive for a total of bounding boxes with a Jaccard index larger than 0.5; *FP* is false positive for a total bounding boxes with a Jaccard index smaller than 0.5; and *FN* is false negative for a total of the images without a correctly detected bounding box. Note that the true negative is set to be 0 according to (23).

## Results

### Ablation for Pre-Processing

**Figure 4:**
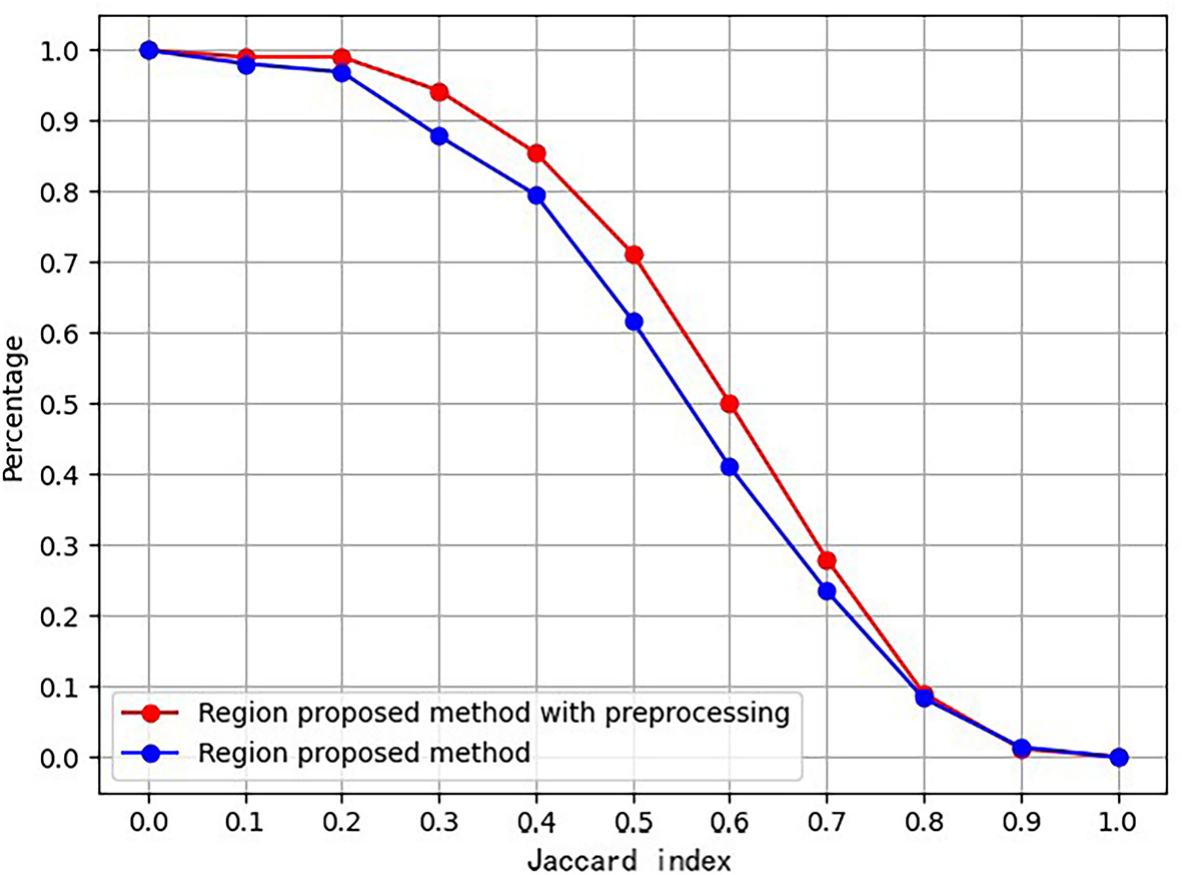
Ablation study to prove effect of proposed preprocessing procedure.

An ablation study was conducted to prove the effect of the proposed preprocessing method. **Figure 4** is the ablation study result. The vertical axis is the ratio of images, whose Jaccard index measuring RoI and ground truth exceeds the corresponding abscissa value.

Furthermore, **Table 1** lists the numbers of RoIs achieved by the region proposal method with and without the preprocessing procedure. The results suggest that the proposed preprocessing procedure can reduce a total of RoIs and save the subsequent computation cost.

**Table 1:** Statistics of RoIs achieved by the region proposal method with and without the preprocessing procedure.

	Region proposal method	Region proposal method
	without preprocessing	with preprocessing
Number of RoIs	840.61 ± 300.24	676.61 ± 215.93
(mean ± std)		

### RoI Extraction and Bounding-Box Regression Results

**Figure 5:**
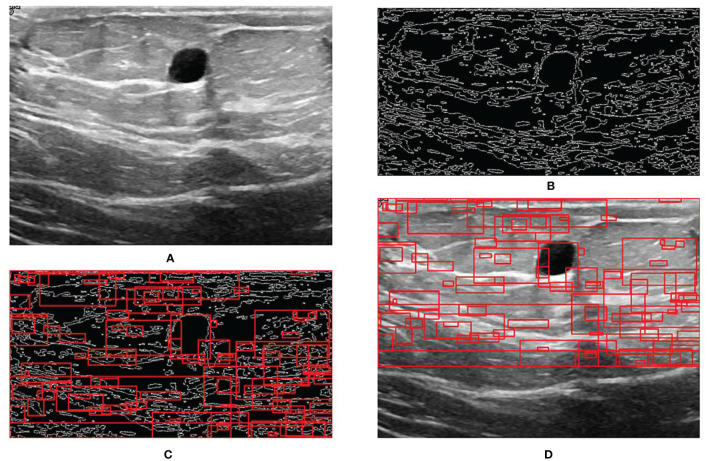
Step-by-step process of RoI extraction. **(A)** Original image. **(B)** Edges obtained by Canny edge detector. **(C)** RoIs obtained by selective search. **(D)** RoIs on the original image.

**Figure 6:**
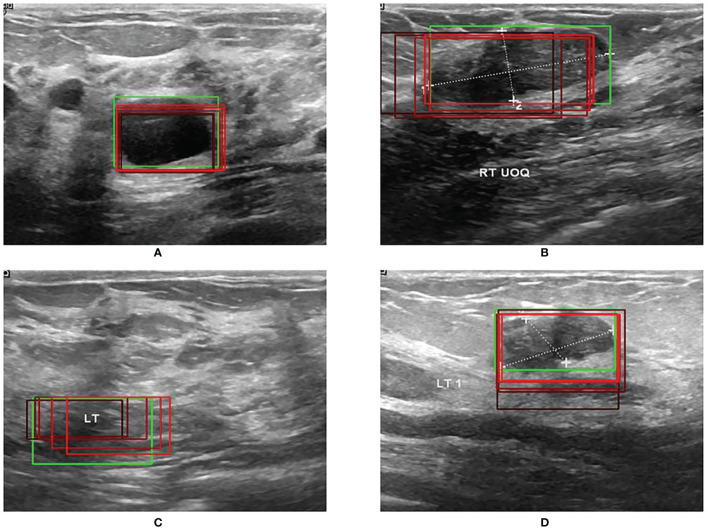
**(A–D)** Four examples of bounding-box regression iterations.

**Figure 5** shows the results of RoI extraction, in which (**A**) is the original image, (**B**) is the edges obtained by the Canny edge detector, (**C**) is the RoIs obtained by selective search, and (**D**) is the RoIs on the original image. **Figure 6** shows the results of the bounding-box regression for four samples. The green box is the ground truth in each sub-figure, and the red gradient boxes are the bounding-box regression results. The darkest red box is the initial bounding box, and the lightest red box is the final iteratively regressed bounding box. The results suggest that the iteration method can constantly extend the intersection area of the regressed box and the ground truth.

### Prediction Performance

The proposed BUSnet was testified by the dataset in (22). The two-stage methods included Faster R-CNN (26) and CASCADE R-CNN (27), and the single-stage method included RetinaNet (28), YoLo V2 (29), and a single-shot detector (30). Note that these methods have been proven to be effective to objection detection in US images. **Table 2** illustrates the performance metrics for the six methods, in which the best values are bolded. The results prove the excellent performance of the proposed BUSnet.

**Table 2:** Performance metrics for six methods.

Tumor type	Method	IoU (mean ± std)	accuracy	precision	recall	*F*_1_ score
Benign	BUSnet	**0.566 ± 0.209**	**0.651**	0.651	**1.000**	**0.789**
	Faster R-CNN	0.540 ± 0.266	0.538	**0.832**	0.603	0.699
	CASCADE R-CNN	0.502 ± 0.268	0.489	0.650	0.664	0.657
	Retina Net	0.250 ± 0.121	0.242	0.255	0.821	0.389
	YoLo V2	0.044 ± 0.023	0.020	0.020	0.966	0.039
	SSD300	0.500 ± 0.176	0.139	0.611	0.153	0.245
Malignant	BUS R-CNN	**0.521 ± 0.210**	**0.579**	**0.629**	**0.880**	**0.734**
	Faster R-CNN	0.467 ± 0.212	0.391	0.572	0.553	0.562
	CASCADE R-CNN	0.484 ± 0.230	0.477	0.572	0.741	0.646
	Retina Net	0.295 ± 0.119	0.197	0.207	0.798	0.329
	YoLo V2	0.023 ± 0.011	0.020	0.020	0.956	0.039
	SSD300	0.396 ± 0.243	0.089	0.571	0.095	0.163

Numbers in bold indicate the best result among the listed models.

**Figure 7:**
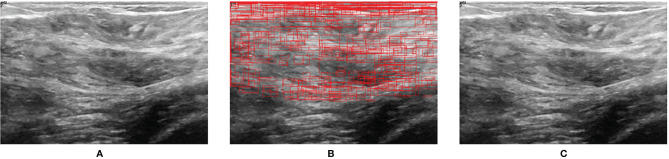
Prediction for a normal US image. **(A)** Original image. **(B)** RoIs obtained after selective search. **(C)** Final output.

**Figure 7** shows the prediction result when a normal US image is inputted into BUSnet. No lesion box is identified in the output.

## Discussion

US imaging is an effective technique for breast cancer screening. This paper proposes a deep learning model, BUSnet, to automatically detect the breast tumor lesions in US images. According to the characteristics of breast US images, the preprocess procedure and the iterative bounding-box regression were integrated in the proposed BUSnet, which were proved to be able to the improve the detection accuracy of lesions.

By far, ML and DL methods have been widely investigated to develop the AI-based US diagnosis techniques. Like the Bayesian network (31) and support vector machine (32), the classic ML methods were used as the classifier in two-stage lesion detection methods (14). proved that the DL methods can achieve better performance than the ML methods for breast US lesion detection. The well-developed DL models, like Faster R-CNN and YoLo, have been applied to breast US lesion detection (33). However, AI research and development for US images are still slow, compared with other modalities (4).

To improve the performance of DL-based lesion detection for breast US images, the preprocessing, the classification and bounding-box regression, and the post-processing should be integratedly considered. The imaging quality problems in US, like speckle noise, can disturb the classification of RoIs. Thus, the preprocessing procedure included the histogram equalization, normalization, and wavelet domain denoising. Because most breast tumors are located in the upper part of the US images (24), we cut out 30% of the area under the US images in the preprocessing procedure to save the subsequent computation burden. After preprocessing, we adopted a two-stage approach, including the region proposal and bounding-box regression, for the lesion detection. To effectively extract RoIs in the US images, we combined the Canny edge detector with the selective search in the region proposal stage. Because the ultrasound image is monochrome, the edge information is very important for detecting the lesion area. Thus, we first used a Canny edge detector to capture edges in images. Note that a Canny edge detector captures a huge number of edges in images. To tackle this problem, the selective search is used after the Canny edge detector to decrease the redundant edges. The RoIs obtained by selective search can reinforce the features of lesion for the further detection. According to (25), ResNet50 showed outstanding performance for the breast US images and acted as the backbone network for the classification of RoIs and bounding-box regression in the proposed BUSnet. The aggregation was applied as the post-processing procedure, which was proven to be able to exclude the background of US images from the lesions effectively. Our method discarded the bounding boxes identified as lesions with the probability less than 0.9. Thus, when a normal image is inputted into BUSnet, BUSnet will output no lesion box in the image.

The experimental results indicate that the proposed BUSnet worked well as expected. The ablation study shown in **Figure 4** confirms the proposed preprocessing procedure. **Figure 6** proves that the iteration regression strategy helped to continuously improve the bounding boxes. The comparison between our proposed BUSnet and other advanced methods is shown in **Table 2**. Overall, all the two-stage approaches, including our BUSnet, Faster R-CNN, and CASCADE R-CNN, performed better than the one-stage approaches, including Retina Net, YoLo V2, and SSD300. Furthermore, BUSnet achieved better performance than the other two-stage methods.

In this research, the dataset size is still small. Extending the capacity of the labeled dataset will be a crucial issue in the future. Furthermore, the semi-supervised learning and the transfer learning strategies will be considered for BUSnet for the incompletely labelled datasets and the various fields collected datasets, respectively.

## Conclusions

US is an effective modality for the breast cancer screening. This paper proposed a deep learning model called BUSnet for the lesion detection in US images. BUSnet integrates preprocessing, region proposal, bounding-box regression, and post-processing. BUSnet can achieve satisfying lesion detection accuracy, which can be further applied to develop AI-based diagnostic support technologies for breast disease screening.

## Data Availability Statement

Publicly available datasets were analyzed in this study. These data can be found as follows: https://scholar.cu.edu.eg/?q=afahmy/pages/dataset.

## Author Contributions

HG and HW conceived the idea for this study. YL worked on the end-to-end implementation of the study. JW provided relevant insights on the clinical impact of the research work and handled the redaction of the paper. PQ managed the project and provided the funding for the research. All authors contributed to the article and approved the submitted version.

## Funding

This work was supported by the National Natural Science Foundation of China (grant number 61633006 and 81872247) and the Fundamental Research Funds for the Central Universities, China (grant number DUT21YG118).

## Conflict of Interest

The authors declare that the research was conducted in the absence of any commercial or financial relationships that could be construed as a potential conflict of interest.

## Publisher’s Note

All claims expressed in this article are solely those of the authors and do not necessarily represent those of their affiliated organizations, or those of the publisher, the editors and the reviewers. Any product that may be evaluated in this article, or claim that may be made by its manufacturer, is not guaranteed or endorsed by the publisher.
